# Downregulation of sST2, a decoy receptor for interleukin‐33, enhances subcutaneous tumor growth in murine pancreatic cancer cells

**DOI:** 10.1002/2211-5463.70099

**Published:** 2025-08-02

**Authors:** Miho Akimoto, Nobuko Koshikawa, Takao Morinaga, Mimi Tamamori‐Adachi, Atsushi Takatori, Keizo Takenaga

**Affiliations:** ^1^ Department of Biochemistry Teikyo University School of Medicine Tokyo Japan; ^2^ Division of Innovative Cancer Therapeutics Chiba Cancer Center Research Institute Japan; ^3^ Division of Cell Therapy Chiba Cancer Center Research Institute Japan

**Keywords:** cancer‐associated adipocytes, IL‐33, pancreatic cancer, sST2, subcutaneous tumor growth, tumor‐associated neutrophils

## Abstract

Despite the recent scientific advancements, pancreatic cancer remains the seventh leading cause of cancer‐related mortality. Pancreatic cancer progression is closely associated with inflammation, and we previously showed that short hairpin RNA‐mediated knockdown of sST2 expression, a soluble decoy receptor for the proinflammatory molecule interleukin‐33 (IL‐33), in mouse Panc02 pancreatic cancer cells reduced malignant growth following pancreatic (orthotopic) implantation. Furthermore, this growth suppression was accompanied by decreased tumor angiogenesis, reduced expression of the neutrophil chemoattractant CXCL3, and a lower number of tumor‐associated neutrophils (TANs). In contrast to previous results, in this study, we showed that IL‐33‐dependent tumor growth and pulmonary metastasis occurred following subcutaneous (ectopic) implantation of sST2‐knockdown cells. This was associated with a decrease in the levels of the anti‐inflammatory molecule adiponectin and the number of GLUT4‐positive cancer‐associated adipocytes, as well as an increase in IκBα phosphorylation levels, *Cxcl3* expression, and the accumulation of infiltrating CD206^+^ protumor N2 TANs. Taken together, these results suggest that Panc02 cell‐derived sST2 differentially affects malignant growth in the tumor microenvironment depending on the implantation site.

## Graphical abstract

Subcutaneous implantation of murine Panc02 pancreatic cancer cells depleted of sST2, a soluble decoy receptor for the proinflammatory interleukin‐33 (IL‐33), leads to a decreased number of GLUT4‐positive cancer‐associated adipocytes, reduced levels of the anti‐inflammatory molecule adiponectin, increased phosphorylation of IκBα, elevated Cxcl3 expression, and accumulation of CD206‐positive protumor N2 TANs in the tumor microenvironment, thereby promoting tumor growth and metastasis.

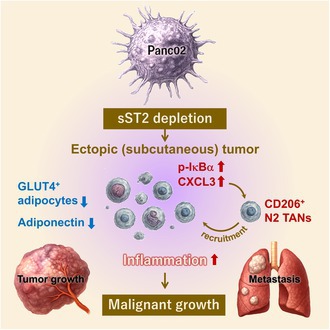

**Abbreviations**
AdipoQ: adiponectin
AMPK: AMP‐activated protein kinase
GLUT4: glucose transporter type 4
IL‐33: interleukin 33
IκBα: NF‐κB‐inhibiting factor α
MPO: myeloperoxidase
NF‐κB: nuclear factor‐κB
RT‐qPCR: reverse transcription‐quantitative polymerase chain reaction
shRNA: short hairpin RNA
TAN: tumor‐associated neutrophil

Pancreatic cancer is the 13th most common cancer worldwide, with over 495 000 new cases diagnosed in 2020. It is one of the most lethal malignancies, with an overall 5‐year survival rate of < 10% [1]. Pancreatic cancer treatment primarily involves cytoreductive surgery, which is suitable only for 10% of patients because most patients present with locally advanced or metastatic disease at diagnosis [2]. Therefore, pancreatic cancer ranks as the seventh leading cause of cancer‐related mortality, resulting in over 466 000 deaths annually [3]. Pancreatic cancer progression is closely associated with inflammation, making a better understanding of the inflammatory tumor microenvironment critical for developing more effective therapeutic approaches.

ST2 belongs to the interleukin‐1 (IL‐1) receptor family [4], and its pre‐mRNA undergoes alternative splicing to produce several isoforms, including sST2 (a soluble form) and ST2L (a transmembrane form) [5, 6, 7]. While ST2L is found on the membranes of various cell types [5, 6, 7], sST2 is predominantly expressed on fibroblasts and epithelial cells [8, 9].

Interleukin‐33 (IL‐33), which belongs to the IL‐1 family [10, 11], is generally localized in the nucleus and released as an alarmin during cell injury or tissue damage [12]. IL‐33 binds to a heterodimeric receptor consisting of ST2L and the IL‐1 receptor accessory protein (IL‐1RAcP) [13], and sST2 disrupts this interaction by acting as a decoy receptor [9, 10]. The IL‐33/ST2L axis activates the production of both pro‐ and anti‐inflammatory cytokines by recruiting myeloid differentiation primary response 88 (MyD88) [14] and subsequently activating nuclear factor‐κB (NF‐κB) signaling through the promotion of TGFβ‐activated kinase (TAK1) binding to TNF receptor‐associated protein 6 (TRAF6), which activates canonical IκB kinases (IKKs) that phosphorylate IκB, leading to its degradation and the release of NF‐κB [15]. Besides its role in inflammation, the IL‐33/ST2L axis has been implicated in cancer progression. For instance, elevated IL‐33 serum levels are positively correlated with poor prognosis in various cancer types [16, 17, 18]. The IL‐33/ST2L axis promotes tumor progression in a mouse model of breast cancer by suppressing innate antitumor immunity through the accumulation of immunosuppressive cells [19]. We previously demonstrated in a Lewis lung carcinoma (3LL) model that IL‐33 induces the death of ST2L‐positive weak metastatic cells but not of ST2L‐negative highly metastatic cells, suggesting the selection of highly metastatic cells within the tumor microenvironment [20]. We also demonstrated that sST2 suppresses the growth and metastasis of mouse and human colon carcinoma cells [21, 22]. Additionally, hypoxia, which promotes malignant behavior, including invasion, metastasis, angiogenesis, and chemoresistance, was found to decrease sST2 expression via the hypoxia‐inducible factor (HIF)‐nuclear IL‐33‐GATA3 pathway [23]. These findings indicate that IL‐33 and sST2 exert protumor and antitumor effects, respectively. In contrast, IL‐33 exhibits antitumor properties in B16 melanoma, 3LL, and 4 T1 mammary tumor models by enhancing the cytotoxicity and tumor infiltration of cytotoxic T cells and natural killer cells [24, 25]. We also found that short hairpin RNA (shRNA)‐mediated downregulation of sST2 in mouse Panc02 pancreatic cancer cells suppresses the growth of sST2‐knockdown cells in the pancreas through a reduction in the levels of the neutrophil chemoattractant CXCL3 and the number of tumor‐associated neutrophils (TANs) in the tumor microenvironment, indicating the antitumor and protumor activities of IL‐33 and sST2, respectively [26]. The modulation of the IL‐33/ST2L axis by sST2 in the tumor microenvironment clearly impacts tumor growth; however, its effect is likely cell‐type‐specific and context‐dependent.

Tumor‐associated macrophages (TAMs) comprise two polarizable subtypes—M1 TAMs, which suppress cancer progression, and M2 TAMs, which promote invasion, metastasis, and chemoresistance [27]. Both subtypes express myeloperoxidase (MPO) and the pan‐macrophage marker CD68, and M2 TAMs specifically express CD163 and CD206 [27]. Similarly, TANs are classified as antitumor N1 and protumor N2 neutrophils. Typical markers for N1 TANs are MPO^+^CD11b^+^CD206^−^ and CD45^+^Ly‐6G^+^CD206‐, while those for N2 TANs are MPO^+^CD11b^+^CD206^+^ and CD45^+^Ly‐6G^+^CD206^+^ [28, 29, 30, 31]. N1 TANs participate in the antibody‐dependent or direct killing of tumor cells [31]. N2 TANs are involved in tumor angiogenesis, matrix remodeling, and suppression of cytotoxic T lymphocyte function [32], which correlates with poor prognosis and disease progression in patients with various cancers [28, 29].

The behavior of tumor cells is influenced by whether the implantation site is orthotopic or ectopic (usually subcutaneous). For example, compared with the orthotopic microenvironment, the subcutaneous microenvironment induces significant changes in the gene expression profile of human pancreatic cancer cells [33], chemosensitivity of murine colon carcinoma cells [34], invasive phenotype of *ras*‐transfected human bladder cancer cells [35], tumor growth of breast cancer patient‐derived xenografts [36], and tumor vasculature of human breast cancer xenografts and a mouse K‐*ras*‐induced lung cancer model [37, 38]. In this study, we examined how the behavior of sST2‐knockdown Panc02 cells differs when implanted subcutaneously versus orthotopically. We found that compared with orthotopic tumor growth, subcutaneous implantation resulted in significantly increased tumor growth, accompanied by downregulation of anti‐inflammatory adiponectin (AdipoQ) expression, activation of NF‐κB, upregulation of *Cxcl3* expression, and accumulation of N2 TANs. These results highlight the importance of the tumor microenvironment in determining the role of the IL‐33/ST2L axis in the growth of pancreatic cancer and possibly other cancers.

## Materials and methods

### Reagent

Murine recombinant adiponectin (rAdipoQ) was purchased from PeproTech (Rocky Hill, NJ, USA) for *in vitro* experiments and Oriental Yeast (Tokyo, Japan) for *in vivo* experiments.

### Cells and cell culture

The establishment and characterization of shRNA‐mediated ST2‐knockdown Panc02 cells—particularly, Panc02‐sh#3 cells (downregulating both ST2L and sST2) and Panc02‐sh#5 cells (downregulating only sST2)—as well as the control Panc02‐shCont cells (Panc02‐shCont) were previously described [26]. These cells were cultured in Dulbecco's modified Eagle's medium supplemented with 10% fetal bovine serum and 1% penicillin–streptomycin (FUJIFILM Wako Pure Chemical Corp., Osaka, Japan) in a humidified atmosphere containing 21% O_2_ and 5% CO_2_ at 37 °C. All cell lines were free of mycoplasma contamination, as confirmed using an e‐Myco Mycoplasma PCR Detection Kit (Cosmo Bio Co. Ltd., Tokyo, Japan).

### RNA preparation and RT–qPCR

Total RNA was extracted from cells and tumor tissues using the RNeasy Plus Mini Kit (QIAGEN, Hilden, Germany) and TRI reagent (Sigma–Aldrich, Merck, St. Louis, MO, USA), respectively, and 1 μg of total RNA was reverse transcribed using oligo(dT) primers and M‐MLV reverse transcriptase (Invitrogen, ThermoFisher Scientific, Waltham, MA, USA) or ReverTra Ace qPCR RT Master Mix (TOYOBO, Tokyo, Japan). The synthesized cDNA was subjected to quantitative PCR, as previously described [26]. Relative gene expression levels were determined using the 2^−ΔΔ*C*^_*t*_ method and normalized to that of *Gapdh*. PCR primer sequences are detailed in Table S1.

### Tumor implantation

Animal experiments were performed with the approval of the Committee on the Ethics of Animal Experiments of Chiba Cancer Center (permission number: 18–1) and in accordance with the guidelines of the Institutional Review Board of Teikyo University (permission number: 22–020 and 24–016). The experiments adhered to the institutional guidelines for the care and use of research animals, the ARRIVE2.0 guidelines, and the EU Directive 2010/63/EU for animal experiments. Panc02 cells and their variants (2 × 10^5^ cells) were subcutaneously implanted into 6–7‐week‐old syngeneic male C57BL/6N mice (CLEA Japan, Tokyo, Japan) or C57BL/6N‐IL‐33^−^/^−^ mice (CDB0631K; IL‐33 KO mice) [26, 39] (RIKEN CDB, Kobe, Japan; https://large.riken.jp/distribution/mutant‐list.html). These mice were bred in our laboratory animal facility. Mice were examined for their health by monitoring activity, body weight, food/water intake, and coat/skin condition every 2 days during tumor growth following tumor transplantation. Blistering or ulceration of tumors was not observed by the end of the experiments. In addition, no mice showed severe signs of illness such as lethargy following tumor formation. The maximum tumor size allowed was 10% of the animal's body weight, and animals were monitored throughout the experiment to ensure that tumor size did not exceed this threshold. In this study, no animals were euthanized due to excessive tumor size, as the maximum tumor weight expressed as a percentage of body weight was less than 6.5%. The tumor volume (*V*) was determined by measuring two perpendicular diameters with calipers and using the eq. *V* = (*a* ^2^ × *b*)/2, where *a* is the small diameter, and *b* is the large diameter. Mice were euthanized via CO_2_ inhalation at the study's conclusion. Animal death was confirmed by respiratory arrest and verifying no heartbeat by palpation. The primary tumors were resected at necropsy for tumor weight measurement. Lungs were removed and fixed in Bouin's solution, and the parietal metastatic nodules were counted.

### Intratumor administration of rAdipoQ

C57BL/6N mice were subcutaneously injected with 2 × 10^5^ Panc02‐sh#5 cells, and tumors were allowed to grow to an approximate volume of 0.1 cm^3^. Next, the mice were randomly assigned to groups, with 7 mice in each group, and received an intratumoral injection of 5 μg rAdipoQ in 50 μL Dulbecco's phosphate‐buffered saline (DPBS) per tumor on Day 0 and 6.5 μg rAdipoQ in 100 μL DPBS per tumor on Day 4. Tumor growth was monitored with caliper measurements performed every 3 days, as described above. The tumors were resected and weighed at the end of experiments.

### Matrigel invasion assay

The invasive abilities of Panc02‐shCont, Panc02‐sh#3, and Panc02‐sh#5 were assessed using an impedance‐based xCELLigence RTCA DP instrument (Agilent Technologies, Santa Clara, CA, USA). Filters were coated with 20 μL of a Corning Matrigel basement membrane matrix (diluted at 1 : 40). Cells (4 × 10^4^) suspended in 100 μL of DMEM and 160 μL of DMEM supplemented with 10% FBS were added to the upper and lower chambers, respectively. The instrument was placed in a CO_2_ incubator, and the invasiveness of the cells was monitored for up to 18 h.

### Profiling chemokine and cytokine gene expression

Differentially expressed chemokine and cytokine genes in Panc02‐shCont and Panc02‐sh#5 tumors were analyzed using a Mouse Cytokines & Chemokines RT2 Profiler PCR Array (QIAGEN, Hilden, Germany).

### Western blotting

Tumor tissues without necrotic areas were cut into pieces using a razor blade, and proteins were extracted using RIPA buffer (50 mm Tris–HCl [pH 7.4], 150 mm NaCl, 1% NP‐40, 0.5% deoxycholate, 0.1% sodium dodecyl sulfate, and 2 mm EDTA), containing cOmplete protease inhibitor cocktail and PhosSTOP phosphatase inhibitor cocktail (Roche Applied Science, Penzberg, Germany) on ice for 30 min. The lysates were centrifuged at 15000 × ***g*** for 10 min at 4°C, and the supernatants were subjected to western blotting. Protein concentrations were measured using the Bradford method with bovine serum albumin as the standard. Mouse monoclonal anti‐AdipoQ (#ab22554; 19F1; Abcam, Cambridge, UK), rabbit monoclonal anti‐IκBα (#4812; 44D4; Cell Signaling Technology, Danvers, MA, USA), rabbit monoclonal anti‐phospho‐IκBα (Ser32) (#2859; 14D4; Cell Signaling Technology), and mouse monoclonal anti‐β‐actin (C4) (#sc‐47 778; Santa Cruz Biotechnology, Dallas, TX, USA) were used as primary antibodies. HRP‐conjugated goat anti‐rabbit IgG and HRP‐conjugated goat anti‐mouse IgG (both from Cell Signaling Technology) were used as secondary antibodies. Signals were visualized using ECL Plus (GE Healthcare, Little Chalfont, UK), and the membranes were scanned using a LAS4000 Lumino Imaging Analyzer (GE Healthcare). Uncropped western blot images are shown in Fig. S9.

### Immunohistochemistry

Tumor tissues were harvested and frozen in optimal cutting temperature compound or fixed in 4% paraformaldehyde and embedded in paraffin. For single, double, or triple immunostaining of ST2, CD31, alpha smooth muscle actin (α‐SMA), AdipoQ, glucose transporter type 4 (GLUT4), and CD90 and for double immunostaining of MPO and CD206, cryostat sections were fixed in 4% formaldehyde, permeabilized with 0.5% Triton X‐100 in Dulbecco's phosphate‐buffered saline (DPBS) for 3 min, blocked with 1% bovine serum albumin in DPBS, and then subjected to immunostaining as previously described [26]. For immunostaining of MPO and CD68, deparaffinized sections were incubated in DAKO REAL target retrieval solution (S2031; Agilent Technologies) at 120 °C for 10 min for antigen retrieval, immersed in 3% H_2_O_2_ prepared in DPBS for 30 min at 25 °C to inactivate endogenous peroxidase, and treated with protein block serum‐free solution (Dako) overnight at 4 °C to block nonspecific binding sites. Rabbit polyclonal anti‐ST2 (#11920‐1‐AP; Proteintech, Rosemont, IL, USA), rat monoclonal anti‐CD31 [MEC 13.3] (#550274; BD Pharmingen, Franklin Lakes, NJ. USA), mouse monoclonal anti‐AdipoQ [19F] (Abcam), rabbit polyclonal anti‐α‐SMA (#ABT1487; Sigma‐Aldrich, Merck), FITC‐conjugated rat monoclonal anti‐mouse CD90.2 (Thy1.2) [53–2.1] (#140303; BioLegend, San Diego, CA, USA), rabbit polyclonal anti‐GLUT4 [H‐61] (#sc‐7938; Santa Cruz Biotechnology), rabbit monoclonal anti‐MPO [EPR20257] (#ab208670; Abcam), rabbit monoclonal anti‐CD68 [E3O7V] (#97778; Cell Signaling Technology), and rat monoclonal anti‐mouse CD206‐RPE [MR5D3] (#MA5‐16872; BIO‐RAD, Hercules, CA, USA) antibodies were used as primary antibodies. Alexa Fluor 488‐, Alexa Fluor 594‐, and Alexa Fluor 633‐conjugated species‐specific antibodies were used as secondary antibodies. Nuclei were stained with DAPI (1 μg·mL^−1^) or hematoxylin. The slides were then mounted and observed under an all‐in‐one fluorescence microscope (BZ‐X810; Keyence, Osaka, Japan) or bright‐field microscope. The tumor vessel density was determined by estimating the pixel values of CD31‐positive areas in each image using imagej software (National Institutes of Health), as previously described [26].

### Fluorescence‐activated cell sorting

For fluorescence‐activated cell sorting (FACS) of single cells isolated from tumors and spleens, the tissues were first cut into small pieces using a razor blade. Next, the spleens were minced by crushing with the flat end of the plunger on a 70‐μm nylon cell strainer (BD Biosciences, Franklin Lakes, NJ, USA) and the cells were recovered from the strainer by washing with DMEM supplemented with 10% FBS. The tumors were digested with 0.052 WÃ¼nsch units·mL^−1^ Liberase™ Research Grade (#05401119001; Roche) and 20 μg·mL^−1^ DNase I (#DN‐25; Sigma‐Aldrich, Merck) in DMEM containing 10% FBS for 1 h at 37 °C, with agitation at 50 rpm on a seesaw shaker and pipetting every 15 min. The cell suspension was filtered through a 70‐μm cell strainer, followed by filtration through a 40‐μm cell strainer. Red blood cells were removed from the single‐cell tumor and spleen suspensions using the Red Blood Cell Lysis Buffer (#11814389001; Roche), and the cells were resuspended in the Cell Staining Buffer (BioLegend). Next, the cells were incubated with rat monoclonal anti‐mouse CD16/32 (#101301, clone 93; BioLegend) for 5 min at 4 °C to block Fc and washed and incubated with FITC‐rat monoclonal anti‐mouse CD45 (#103107, clone 30‐F11; BioLegend), Alexa Fluor 647‐rat monoclonal anti‐mouse Ly‐6G (#127609, clone 1A8; BioLegend), and PE‐rat monoclonal anti‐mouse CD206 (MMR) (#141705, clone C068C2; BioLegend) antibodies for 30 min at 4 °C. The cells were washed twice with the Cell Staining Buffer and resuspended in 500 μL of the buffer. After the addition of 7‐AAD (#420403; BioLegend), the cells were incubated for 10 min in the dark. The samples were analyzed using a FACSLyric flow cytometer (BD Biosciences) to determine the relative proportions of specific cell types. In all analyses, side scatter area vs. forward scatter area was used to exclude debris, forward scatter area vs. forward scatter height was used to exclude cell doublets, and the 7‐AAD vs. FITC‐CD45‐positive populations were used to identify live cells. Splenocytes were used to identify the proper gate setting for CD45^+^Ly‐6G^+^ neutrophils in single‐cell tumor tissue suspensions. Gating to identify populations positive for Ly‐6G and CD206 in CD45‐positive populations was performed using the relevant fluorescent channel. All data were analyzed using the FlowJo software package (FlowJo; Ashland, OR, USA).

### Statistical analysis

All data are presented as mean ± SD. The statistical significance of differences between datasets was analyzed using an unpaired Student's *t*‐test or one‐way ANOVA with Tukey's honestly significant difference test as indicated in the figure legends. Statistical significance was established at a *P*‐value of < 0.05.

## Results

### sST2 knockdown enhances malignant tumor growth of Panc02 cells after subcutaneous implantation

**Fig. 1:**
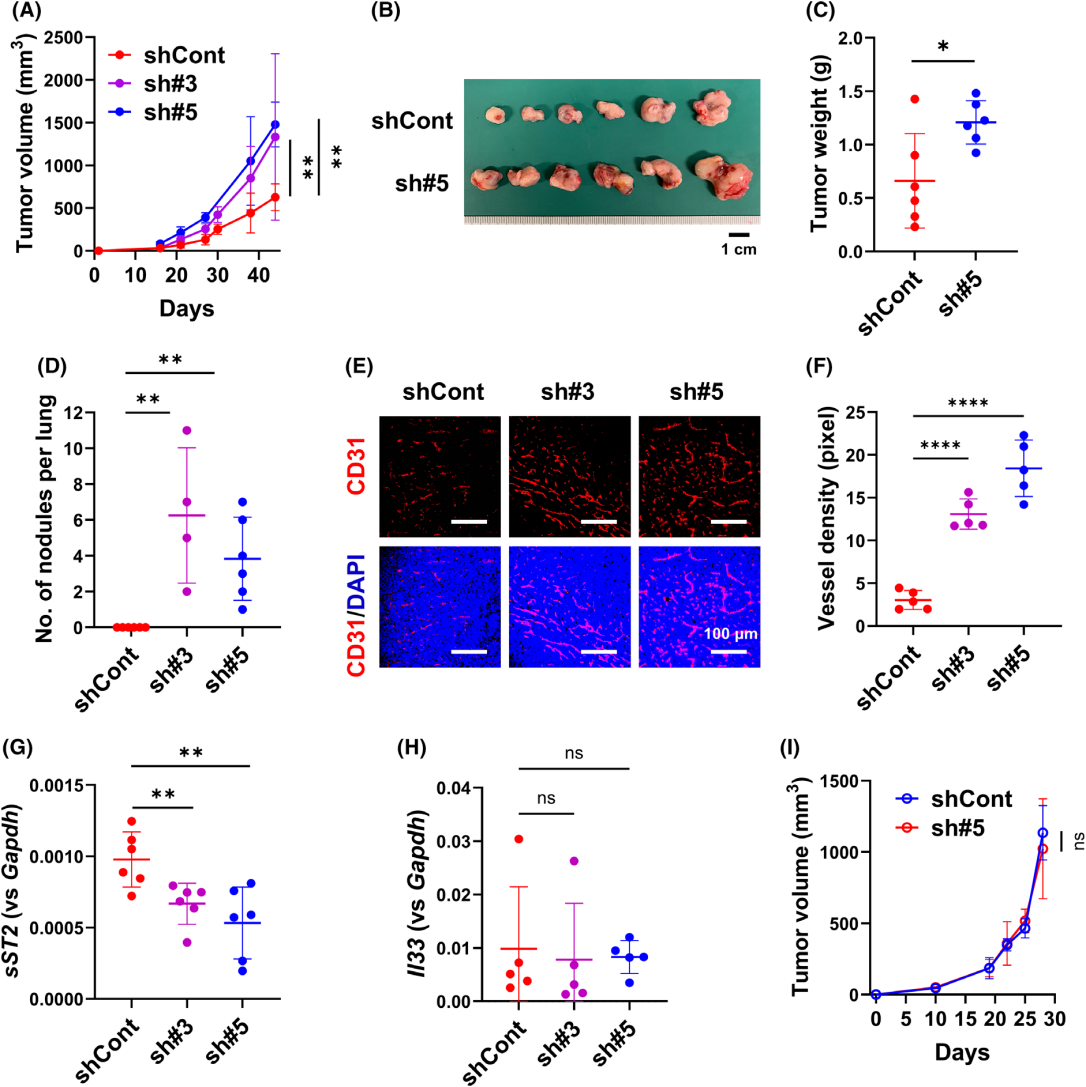
Downregulation of sST2 enhances subcutaneous tumor growth of Panc02 cells. (A) Tumor growth of Panc02‐shCont (shCont) and Panc02‐shsST2 (sh#3 and sh#5) cells. The cells (2 × 10^5^ cells) were subcutaneously implanted into C57BL/6 mice (*n* = 6 mice for shCont and sh#5 and *n* = 4 mice for sh#3). Bars represent mean ± SD. Statistical significance of the difference in tumor size between Panc02‐shCont and Panc02‐sh#5 on Day 43 was evaluated using one‐way ANOVA. (B) Tumors formed by Panc02‐shCont (shCont) and Panc02‐shsST2 (sh#5) cells. Scale bar: 1 cm. (C) Tumor weight. Bars represent mean ± SD. Statistical significance was evaluated using one‐way ANOVA. (D) Number of spontaneous lung metastases derived from the tumors formed by Panc02‐shCont (shCont) and Panc02‐shsST2 (sh#3 ad sh#5) cells (*n* = 4–6 mice per group). Bars represent mean ± SD. Statistical significance was evaluated using one‐way ANOVA. (E) Tumor angiogenesis. CD31 staining of cryostat sections of the tumors formed by Panc02‐shCont (shCont) and Panc02‐shsST2 (sh#3 ad sh#5) cells. Scale bars: 100 μm. (F) Vessel density of tumors formed by Panc02‐shCont (shCont) and Panc02‐shsST2 (sh#3 ad sh#5) cells (*n* = 5 fields). Bars represent mean ± SD. Statistical significance was evaluated using one‐way ANOVA. (G) RT‐qPCR analysis of *sST2* mRNA in Panc02‐shCont (shCont) and Panc02‐shsST2 (sh#3 ad sh#5) tumors. Bars represent mean ± SD. Statistical significance was evaluated using one‐way ANOVA. (H) RT‐qPCR analysis of *IL‐33* mRNA in Panc02‐shCont (shCont) and Panc02‐shsST2 (sh#3 ad sh#5) tumors. Bars represent mean ± SD. Statistical significance was evaluated using one‐way ANOVA. (I) Tumor growth of Panc02‐shCont (shCont) and Panc02‐shsST2 (sh#5) cells in IL‐33 KO mice. The cells (2 × 10^5^ cells) were subcutaneously implanted into C57BL/6N‐IL‐33^−^/^−^ mice (*n* = 6 mice per group). Statistical significance was evaluated using one‐way ANOVA. *P < 0.05, **P < 0.01, ****P < 0.0001, ns: not statistically significant.

*sST2* expression in Panc02‐shCont, Panc02‐sh#3, and Panc02‐sh#5 cells was assessed using RT‐qPCR (Fig. S1), and the growth of subcutaneous tumors derived from these cells was examined. We found that both Panc02‐sh#3 and Panc02‐sh#5 cells developed larger tumors compared to Panc02‐shCont cells (Fig. 1A–C). Panc02‐sh#3 and Panc02‐sh#5 cells metastasized to the lungs, whereas Panc02‐shCont cells did not (Fig. 1D), despite all three cell types exhibiting comparable Matrigel invasive abilities (Fig. S2). Microvessel density, as evaluated using CD31 staining, was significantly increased in Panc02‐sh#3 and Panc02‐sh#5 tumors (Fig. 1E,F). *sST2* expression levels were lower in Panc02‐sh#3 and Panc02‐sh#5 tumors than in Panc02‐shCont tumors (Fig. 1G), corresponding to *sST2* expression at the cellular level. Immunofluorescence staining of the tumor sections using an antibody recognizing both ST2L and sST2 revealed that the ST2 expression was higher in Panc02‐shCont tumors than in Panc02‐sh#3 and Panc02‐#5 tumors. The difference in staining patterns between Panc02‐sh#3 and Panc02‐sh#5 tumors was likely due to differences in ST2L expression (Fig. S3). Due to the lack of commercially available ST2L‐specific antibodies, identifying ST2L‐positive cells other than the tumor cells was challenging; however, we observed a small number of ST2^+^CD90^+^ cells, which were likely group 2 innate lymphoid cells (ILC2s) with protumorigenic activity [19], in Panc02‐shCont tumors (Fig. S3). The abundance of *IL‐33* mRNA did not differ between the sST2‐knockdown and control tumors (Fig. 1H). Moreover, tumor growth of Panc02‐sh#5 cells was comparable to that of Panc02‐shCont cells in IL‐33 KO mice (Fig. 1I). Collectively, these results indicate the involvement of the IL‐33/ST2L axis in promoting tumor growth and the antitumor effects of sST2.

### sST2 knockdown in Panc02 cells downregulates AdipoQ expression

**Fig. 2:**
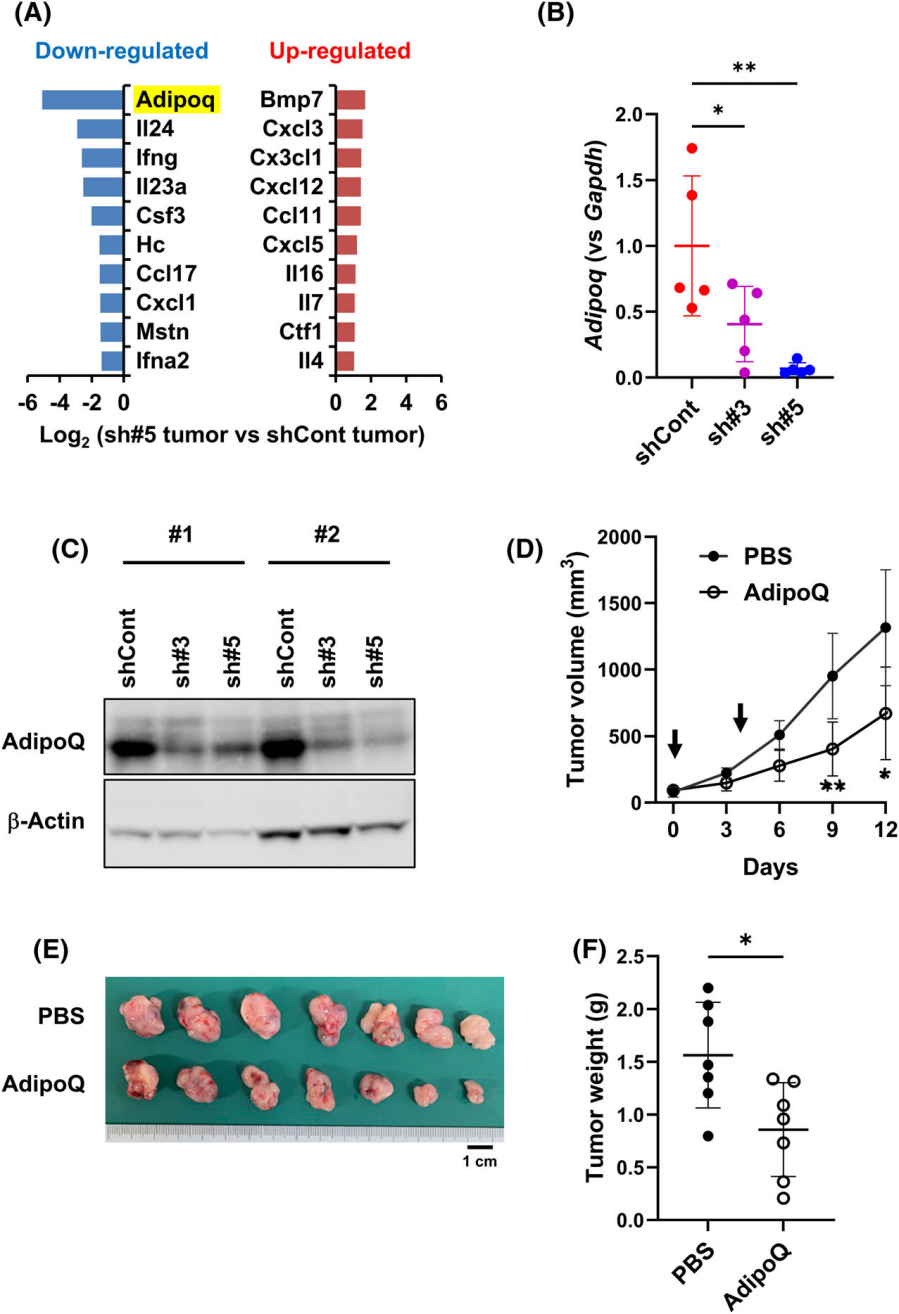
AdipoQ accumulation in Panc02‐shCont and Panc02‐shsST2 tumors. (A) Cytokine and chemokine profiles in Panc02‐shCont and Panc02‐sh#5 cells. (B) RT‐qPCR analysis of *AdipoQ* mRNA expression in Panc02‐shCont and Panc02‐shsST2 tumors (*n* = 5). Bars represent mean ± SD. Statistical significance was evaluated using one‐way ANOVA. *P < 0.05, **P < 0.01. (C) Western blot analysis of AdipoQ expression in Panc02‐shCont and Panc02‐shsST2 tumors (*n* = 2, #1 and #2 tumors). β‐Actin is used as the loading control. Uncropped western blot images are presented in Fig. S9. *P < 0.05, **P < 0.01. (D) Growth of Panc02‐sh#5 tumors after intratumoral injection of AdipoQ. Control group and rAdipoQ group (*n* = 7 mice each) were injected with PBS and rAdipoQ, respectively, on Day 0 and Day 4 indicated by the arrows. Bars represent mean ± SD. Statistical significance was evaluated using one‐way ANOVA. *P < 0.05, **P < 0.01. (E) Resected tumors treated with PBS and AdipoQ. (F) Weight of tumors treated with PBS and AdipoQ. Bars represent mean ± SD. Statistical significance was evaluated using one‐way ANOVA. *P < 0.05.

We compared the expression levels of other cytokines and chemokines between Panc02‐shCont and Panc02‐sh#5 tumors via PCR array analysis and found that *Adipoq* expression was markedly reduced in Panc02‐sh#5 tumors (Fig. 2A, Fig. S4). The reduction in AdipoQ expression was also confirmed at both the mRNA and protein levels in Panc02‐sh#3 and Panc02‐sh#5 tumors (Fig. 2B,C). Next, we intratumorally administered rAdipoQ to determine whether the reduction in AdipoQ was attributable to the enhanced growth of Panc02‐sh#5 tumors. As shown in Fig. 2D–F, the administration of rAdipoQ significantly retarded tumor growth, indicating its antitumor effect.

**Fig. 3:**
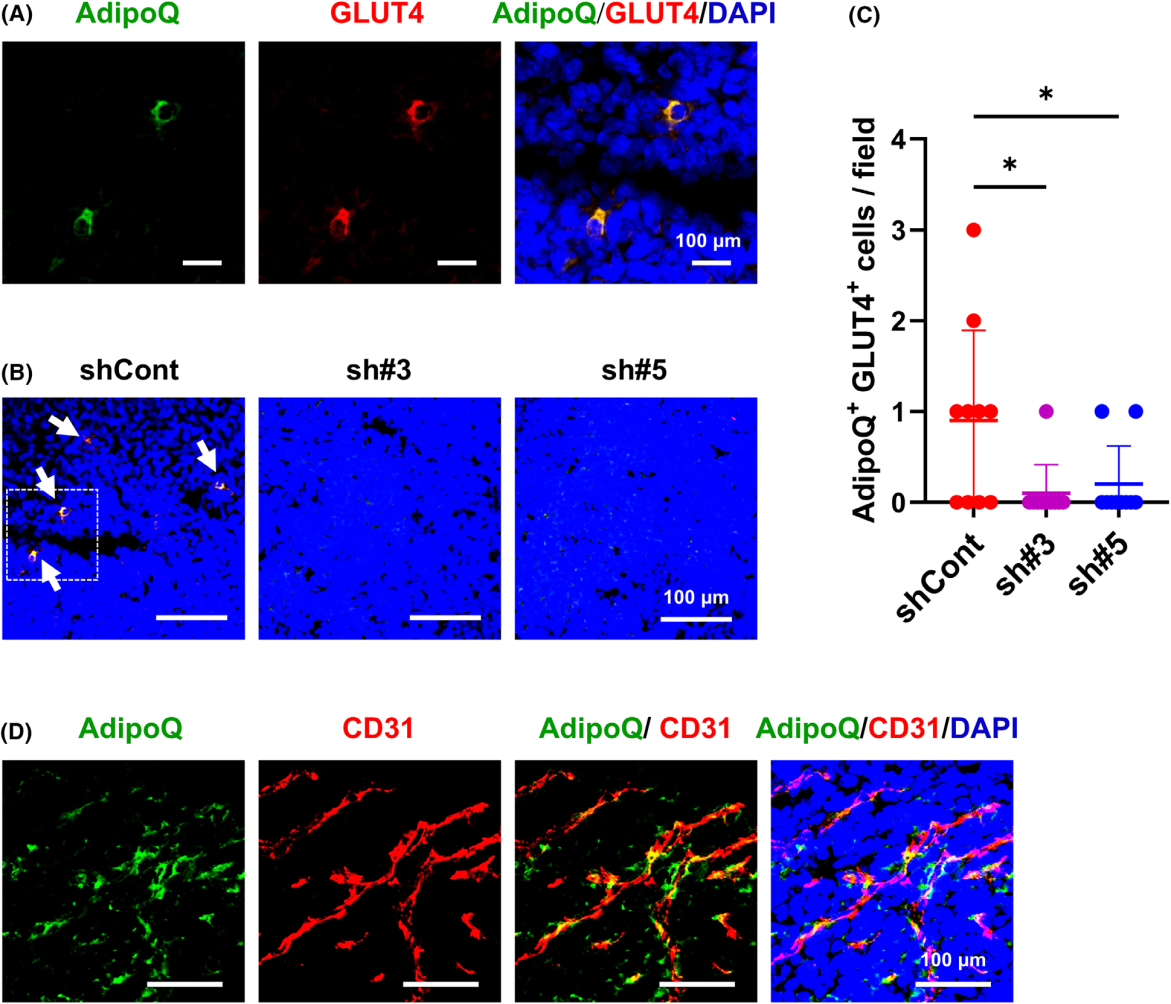
Immunohistochemical analysis of AdipoQ‐expressing cells in Panc02‐shCont and Panc02‐shsST2 tumors. (A) Immunostaining for AdipoQ and GLUT4 in Panc02‐shCont tumors. Scale bar: 100 μm. (B) AdipoQ^+^GLUT4^+^ cells in Panc02‐shCont and Panc02‐shsST2 tumors. Scale bar: 100 μm. The area enclosed by the dotted line is shown in the rightmost image in (A). (C) Number of AdipoQ^+^GLUT4^+^ cells in the fields (*n* = 10). Bars represent mean ± SD. Statistical significance was evaluated using one‐way ANOVA. *P < 0.05. (D) Immunostaining for AdipoQ and CD31 in Panc02‐shCont tumors. Note that AdipoQ^+^ cells are localized near CD31^+^ microvessels. Scale bar: 100 μm.

Immunofluorescence staining of Panc02‐shCont tumor tissues with anti‐AdipoQ antibody revealed the presence of AdipoQ^+^ cells, which also exhibited positive staining for GLUT4, an adipocyte marker protein (Fig. 3A,B) [40, 41]. Consistent with the reduction in AdipoQ expression, fewer AdipoQ^+^GLUT4^+^ cells were observed in Panc02‐sh#3 and Panc02‐sh#5 tumors than in Panc02‐shCont tumors (Fig. 3C). Although the biological relevance of this finding remains unclear, AdipoQ^+^GLUT4^+^ cells were found to typically accumulate near CD31‐positive microvessels in Panc02‐shCont tumors (Fig. 3D). Furthermore, AdipoQ seemed to be colocalized with α‐SMA^+^ cells (Fig. S5A). However, on closer inspection, some AdipoQ^+^ cells were attached to α‐SMA^+^ mural cells but not to CD31^+^ endothelial cells (Fig. S5B,C). In addition, AdipoQ accumulated very close to the mural cells (Fig. S5B,C).

### sST2‐knockdown tumors exhibit a more inflammatory tumor microenvironment accompanied by an accumulation of protumor N2 TANs

**Fig. 4:**
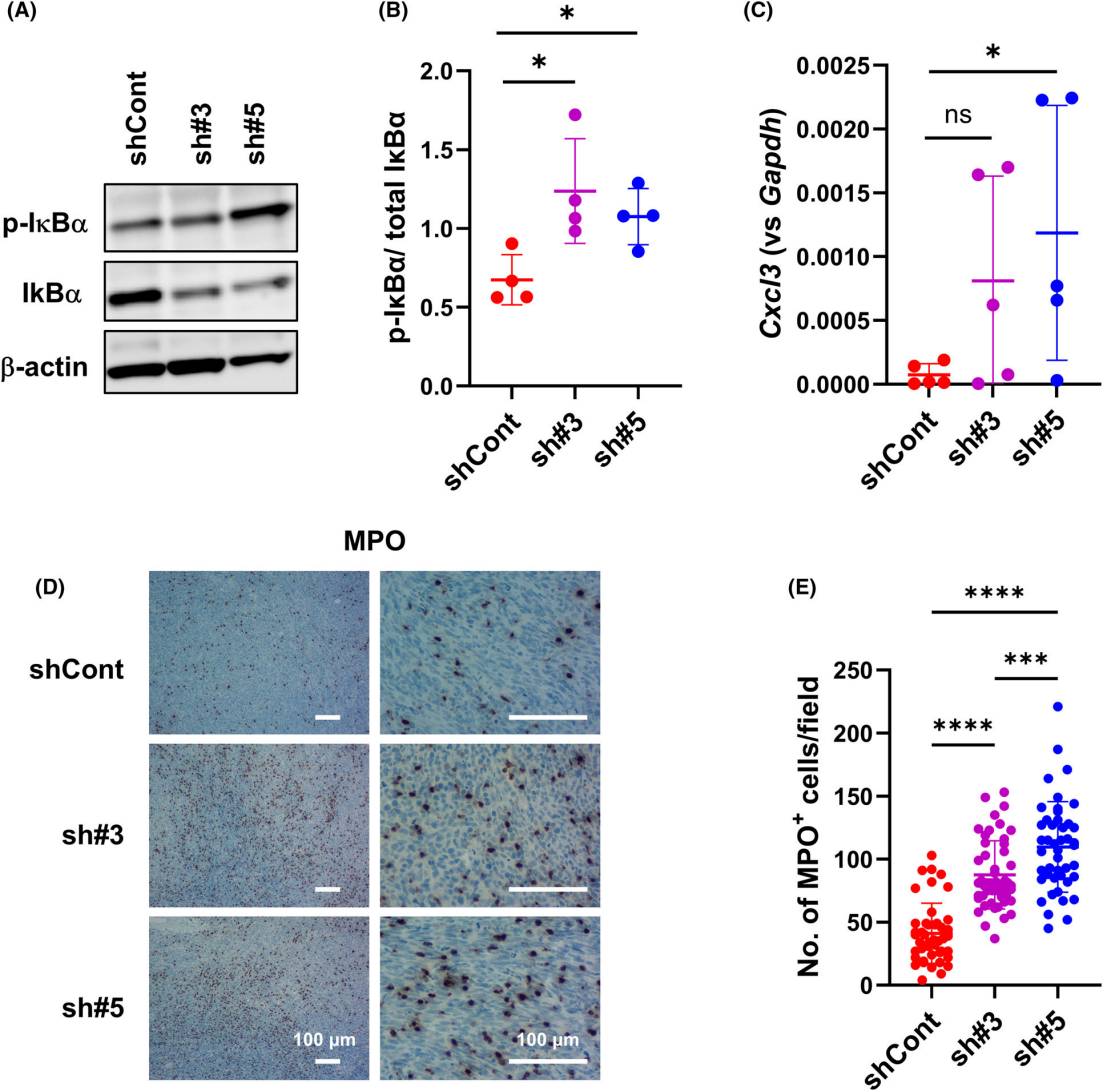
Inflammatory status of Panc02‐shCont and Panc02‐shsST2 tumors. (A) Western blot analysis of IκBα phosphorylation. β‐Actin is used as the loading control. Uncropped images are shown in Fig. S9. (B) Ratio of p‐IκBα to total IκBα. Bars represent mean ± SD. Statistical significance was evaluated using one‐way ANOVA. *P < 0.05. (C) RT‐qPCR analysis of *Cxcl3* mRNA expression. Bars represent mean ± SD. Statistical significance was evaluated using one‐way ANOVA. *P < 0.05, ns: not statistically significant. (D) Low‐ (left) and high‐magnification (right) images of immunohistochemical staining for MPO. Scale bar: 100 μm. (E) Number of MPO‐positive cells per field (*n* = 44, 50, and 45 for shCont, sh#3, and sh#5 tumors, respectively). Bars represent mean ± SD. Statistical significance was evaluated using one‐way ANOVA. ***P < 0.001, ****P < 0.0001.

To investigate the biological role of AdipoQ in Panc02‐shCont tumors, we treated cells expressing the AdipoQ receptors AdipoR1 and AdipoR2 [26] with rAdipoQ and found that it had no influence on the growth and survival of these cells (Fig. S6). As inflammation can promote tumor progression [42] and AdipoQ exerts anti‐inflammatory effects [43], we investigated whether sST2‐knockdown tumors exhibited a heightened inflammatory state compared with that of Panc02‐shCont tumors by assessing the activation of NF‐κB, a master regulator of inflammation and immune homeostasis [44]. We assessed the phosphorylation of IκBα, which triggers proteasomal degradation of IκBα and enables the release of NF‐κB, allowing its translocation into the nucleus [15, 45]. IκBα phosphorylation was elevated in Panc02‐sh#3 and Panc02‐sh#5 tumors compared with that in Panc02‐shCont tumors (Fig. 4A,B), suggesting that sST2‐knockdown subcutaneous tumors are in a more pronounced inflammatory state than Panc02‐shCont tumors.

**Fig. 5:**
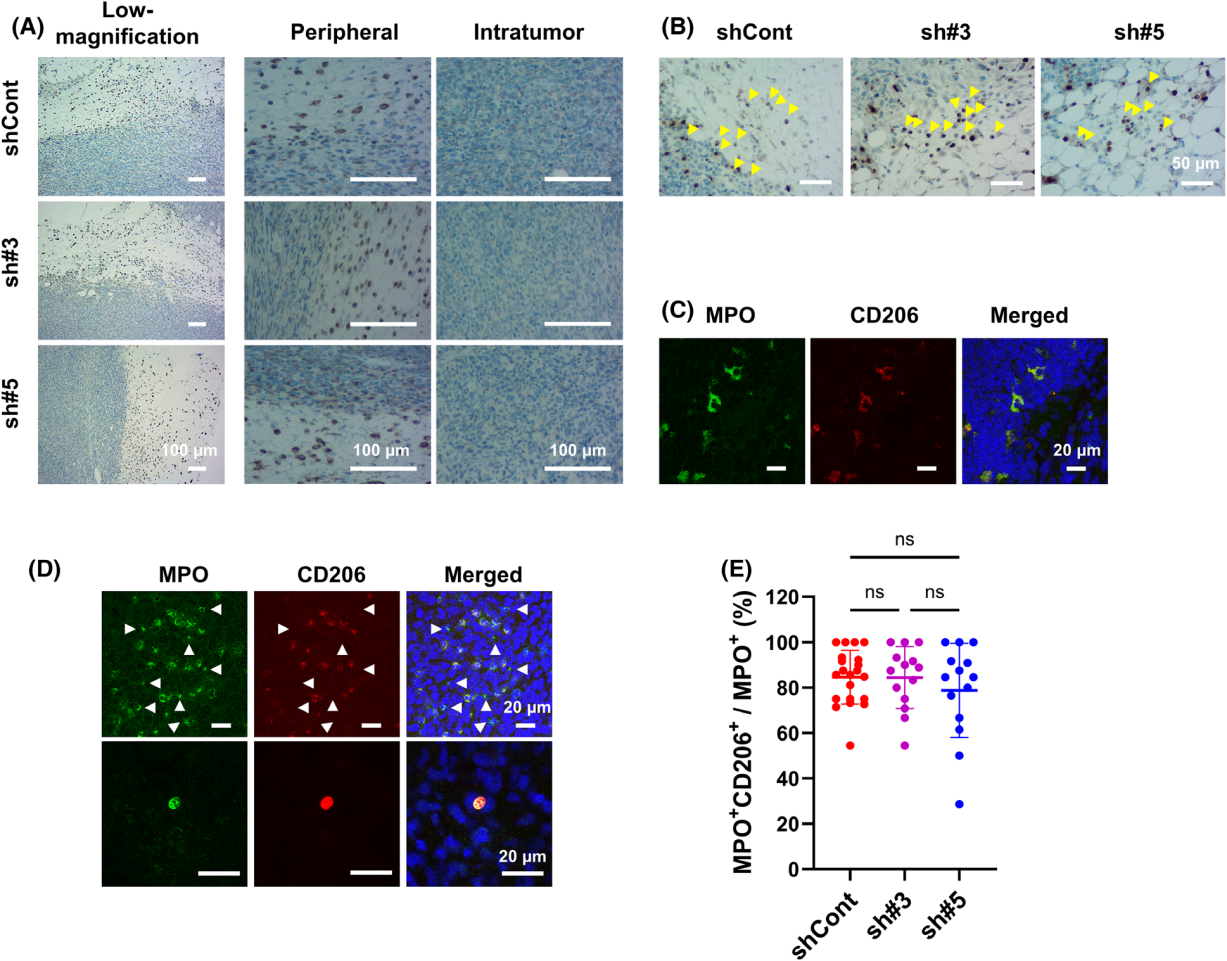
Immunohistochemical analyses of infiltrated cells in Panc02‐shCont and Panc02‐shsST2 tumors. (A) CD68‐expressing cells in the tumors. Low‐ and high‐magnification images of both the peripheral and intratumoral regions are shown. Scale bar: 100 μm. (B) MPO‐expressing cells in the peripheral regions of the tumors. The yellow arrowheads indicate examples of mononuclear MPO^+^ cells. Scale bar: 50 μm. (C) Double immunofluorescence staining for MPO and CD206 in the peripheral regions of the tumors. Scale bar: 20 μm. (D) Double immunofluorescence staining for MPO and CD206 in the intratumor regions. Upper panels: MPO^+^CD206^−^ cells (indicated by arrows) and MPO^+^CD206^+^ cells. Lower panels: Typical MPO^+^CD206^+^ neutrophils with segmented nuclei. Scale bar: 20 μm. (E) Percentage of the number of MPO^+^CD206^+^ cells to the total number of MPO^+^ cells per field (*n* = 21, 14, and 14 for shCont, sh#3, and sh#5 tumors, respectively). Bars represent mean ± SD. Statistical significance was evaluated using one‐way ANOVA. ns: not statistically significant.

Meanwhile, the mRNA expression of the neutrophil chemoattractant *Cxcl3* in Panc02‐sh#3 and Panc02‐sh#5 tumors exhibited an increasing trend (Fig. 4C, Fig. S4), but the expression of other notably downregulated or upregulated genes detected using the PCR array did not clearly correlate with the sST2 expression level (Fig. S7). Hence, we assessed MPO^+^ neutrophil infiltration into the tumors and found that the number of MPO^+^ cells was significantly higher in Panc02‐sh#3 and Panc02‐sh#5 tumors than in Panc02‐shCont tumors (Fig. 4D,E). Furthermore, CD68^+^ and MPO^+^/CD206^+^ cells with round nuclei (likely M2 macrophages) were generally localized at the tumor periphery, with some in the intratumoral regions (Fig. 5A–C), suggesting that most of the infiltrating MPO^+^ cells were TANs. Approximately 80% of these MPO^+^ cells were CD206^+^, indicating that they were likely protumor N2 TANs (Fig. 5D). No difference was observed in the ratio of MPO^+^CD206^+^ to MPO^+^CD206^−^ cells among the Panc02‐shCont, Panc02‐sh#3, and Panc02‐sh#5 tumors (Fig. 5E). Furthermore, by FACS analysis, we observed a trend toward an increase in the percentage of Ly‐6G^+^ neutrophils within the CD45^+^ leukocytes in Panc02‐sh#5 tumors compared with Panc02‐shCont tumors. The percentage of CD206^+^ N2 TANs within the Ly‐6G^+^ neutrophils was not different between Panc02‐shCont and Panc02‐sh#5 tumors (Fig. S8), in general agreement with the immunohistochemical findings.

## Discussion

We demonstrated that subcutaneous injection of sST2‐knockdown Panc02 cells (sh#3 and sh#5) into syngeneic mice enhanced tumor growth, metastasis, and angiogenesis. However, this increased tumor growth was not observed in IL‐33 KO mice. These results indicate that sST2 plays an antitumor role. Moreover, *Cxcl3* expression was increased in sST2‐knockdown tumors. These changes contrast sharply with those observed after orthotopic implantation [26]. Key characteristics of sST2‐knockdown subcutaneous tumors observed were increased tumor angiogenesis, reduced AdipoQ expression, NF‐κB activation, and N2 TAN accumulation. CXCL3 is known to recruit N2 TANs, which then contribute to tumor angiogenesis [31]. The similarity in the ratio of N1 to N2 TANs between Panc02‐shCont and Panc02‐sST2‐knockdown tumors suggests that sST2 may not influence neutrophil polarization toward N1 or N2 in the tumor microenvironment.

AdipoQ exerts its biological effects by binding to its receptors, AdipoR1 and AdipoR2, and subsequently activating AMPK, p38 mitogen‐activated protein kinase, peroxisome proliferator‐activated receptor α, and NF‐κB [46]. In contrast to the findings of the previous study by Kato *et al*. [47], we previously showed that rAdipoQ had no effect on the proliferation and survival of cultured Panc02‐shCont cells [26], which aligns with our current findings. Consequently, we hypothesize that AdipoQ depletion indirectly increases the growth of subcutaneous sST2‐knockdown Panc02 tumors, and thus, we investigated the anti‐inflammatory effects of AdipoQ [38]. We found that NF‐κB was activated in sST2‐knockdown tumors, as evidenced by IκBα phosphorylation levels, indicating elevated inflammation levels in these tumors. This activation is likely due to the IL‐33/ST2L pathway, which is upregulated by the depletion of both sST2 and AdipoQ. This finding is consistent with observations that AdipoQ‐mediated inflammation typically promotes tumorigenesis and progression in various cancers [42]. Additionally, a recent study showed that IL‐33 treatment in ILC2s activates AMPK, which initiates a feedback mechanism that inhibits IL‐33‐induced phosphorylation of IKKα/β and IκBα. Furthermore, AdipoQ synergistically activates AMPK along with IL‐33 to inhibit IL‐33‐induced activation of the NF‐κB pathway [48]. As expected from these observations, our present results demonstrated that the intratumoral injection of AdipoQ indeed suppressed the growth of Panc02‐sh#5 tumors.

AdipoQ‐expressing cells in Panc02‐shCont subcutaneous tumors were also positive for GLUT4, suggesting that these cells are cancer‐associated adipocytes. Adipocytes are known to contribute to pancreatic cancer development and progression in relation to obesity [49]. Similarly, adipocyte‐derived factors, including HGF and glutamine, accelerate murine and human pancreatic cancer cell proliferation [50, 51]. Furthermore, human pancreatic cancer PANC‐1 cells alter the properties of cocultured adipocytes, endowing them with characteristics of cancer‐associated fibroblasts, thereby increasing the invasiveness, chemoresistance, and epithelial–mesenchymal transition of pancreatic cancer cells [52, 53]. These findings reveal the protumor effects of adipocytes. However, a recent study demonstrated that instead of facilitating adiposity, intratumoral adipocytes in pancreatic ductal adenocarcinoma enable CD8^+^ T‐cell infiltration and elicit antitumor responses *in vivo* [54]. Our study also revealed that intratumor adipocytes, rather than those surrounding the tumor in subcutaneous tissues, were associated with reduced subcutaneous tumor growth of Panc02‐shCont cells compared with that of sST2‐knockdown cells. The reason for the lower number of AdipoQ^+^GLUT4^+^ in sST2‐knockdown tumors than in shCont tumors remains unclear. Possible explanations include a reduction in the levels of chemokines that facilitate the targeted migration of adipocytes into tumors, factors that induce the adipogenic differentiation of mesenchymal stem cells homing to tumor locations, or decreased adipogenic differentiation of cancer stem cells (CSCs) in sST2‐knockdown tumors.

In Panc02‐shCont tumors, we observed that the AdipoQ^+^GLUT4^+^ cells were often near microvessels, with some closely associated with α‐SMA^+^ mural cells, likely pericytes [55]. Furthermore, we observed AdipoQ accumulation in close proximity to the mural cells, which may reflect the previously reported binding of AdipoQ to T‐cadherin expressed by pericytes [55]. Of note, CSCs are able to not only initiate and promote tumorigenesis but also differentiate into a diverse range of cell types located predominantly in perivascular niches in many cancers [56]. Therefore, it is possible that AdipoQ^+^GLUT4^+^ cells might have differentiated from CSCs in Panc02‐shCont tumors.

Human pancreatic cancer cell lines express varying levels of sST2, with certain cell lines exhibiting low expression and others exhibiting high expression [26]. Our previous [26] and the present findings suggest that IL‐33 has varying impacts on sST2‐low and sST2‐high human cell lines in terms of their malignant growth at both orthotopic and ectopic sites, suggesting differential actions of IL‐33 at these sites. Given the rarity of cutaneous metastasis in pancreatic cancer [57], further research is required to investigate how IL‐33 affects malignant growth in organs frequently involved in pancreatic cancer metastasis. Furthermore, as sST2‐knockdown mouse colon carcinoma cells exhibit increased malignant growth at both orthotopic and ectopic sites [21], additional studies are needed to investigate whether these findings apply specifically to pancreatic cancer cells.

## Conflict of interest

The authors declare no conflict of interest.

## Author contributions

KT conceived and designed the project. MA, NK, TM, and KT acquired, analyzed, and interpreted the data. KT and MA wrote the paper, and AT and MTA supervised the research. All authors have read and approved the final version of the manuscript submitted for publication.

## Supporting information


**Fig. S1.** RT‐qPCR analysis of sST2 expression in Panc02‐shCont (shCont) and Panc02‐shsST2 (sh#3 ad sh#5) cells.
**Fig. S2.** *In vitro* Matrigel invasion assay using the xCELLigence instrument.
**Fig. S3.** Immunofluorescent analysis of the ST2 expression in Panc02 tumors.
**Fig. S4.** PCR array analysis of cytokine and chemokine gene expression profiles in Panc02‐shCont and Panc02‐sh#5 subcutaneous tumors.
**Fig. S5.** Localization of AdipoQ near microvessels.
**Fig. S6.** Effect of rAdipoQ on the survival of Panc02‐shCont cells.
**Fig. S7.** RT‐qPCR analysis of the expression of the indicated genes in the indicated subcutaneous tumors.
**Fig. S8.** Flow cytometry analysis of neutrophils in Panc02‐shCont and Panc02‐sh#5 tumors.
**Fig. S9.** Uncropped western blot images for Fig. 2C and Fig. 4A.
**Table S1.** Primers used for RT‐qPCR.

## Data Availability

The data that support the findings of this study are openly available in Dryad at https://doi.org/10.5061/dryad.g1jwstr2n, reference number dryad.g1jwstr2n.
