# Supplementary material for: Downregulation of sST2, a decoy receptor for interleukin‐33, enhances subcutaneous tumor growth in murine pancreatic cancer cells
Source: FEBS Open Bio. 2025 Aug 2;15(12):2031–44. doi: 10.1002/2211-5463.70099 (PMC12667206; doi:10.1002/2211-5463.70099)
Supplement: Supplementary file 1 — Fig. S1. RT‐qPCR analysis of sST2 expression in Panc02‐shCont (shCont) and Panc02‐shsST2 (sh#3 ad sh#5) cells. Fig. S2. In vitro Matrigel invasion assay using the xCELLigence instrument. Fig. S3. Immunofluorescent analysis of the ST2 expression in Panc02 tumors. Fig. S4. PCR array analysis of cytokine and chemokine gene expression profiles in Panc02‐shCont and Panc02‐sh#5 subcutaneous tumors. Fig. S5. Localization of AdipoQ near microvessels. Fig. S6. Effect of rAdipoQ on the survival of Panc02‐shCont cells. Fig. S7. RT‐qPCR analysis of the expression of the indicated genes in the indicated subcutaneous tumors. Fig. S8. Flow cytometry analysis of neutrophils in Panc02‐shCont and Panc02‐sh#5 tumors. Fig. S9. Uncropped western blot images for Fig. 2C and Fig. 4A. Table S1. Primers used for RT‐qPCR. [file FEB4-15-2031-s001.pdf]

Supplementary Information

Downregulation of sST2, a decoy receptor for interleukin-33, enhances subcutaneous tumor growth in murine pancreatic cancer cells

Miho Akimoto[1], Nobuko Koshikawa[2], Takao Morinaga[3], Mimi Tamamori-Adachi[1], Atsushi Takatori[2], Keizo Takenaga[2]

[1]Department of Biochemistry, Teikyo University School of Medicine, 2-11-1 Kaga, Itabashi-ku, Tokyo 173-8605, Japan.

[2]Division of Innovative Cancer Therapeutics, Chiba Cancer Center Research Institute, 666-2 Nitona, Chuoh-ku, Chiba 260-8717, Japan.

[3]Division of Cell Therapy, Chiba Cancer Center Research Institute, 666-2 Nitona, Chuoh-ku, Chiba 260-8717, Japan.

Table S1. Primers used for RT–qPCR.

Gene name	Forward/Reverse	Sequence (5' to 3')	Accession No.
Adipoq	Forward	TGTTCCTCTTAATCCTGCCCA	NM_009605.5
	Reverse	CCAACCTGCACAAGTTCCCTT	
IL33	Forward	GGAAGAAGGTGATGGTGAAC	NM_001164724.2
	Reverse	CCACAACATCGTAAGCCAAG	
Ifng	Forward	TCATGGCTGTTTCTGGCTGT	NM_008337.4
	Reverse	TGGACCTGTGGGTTGTTGAC	
Il24	Forward	GCCCAGTAAGGACAATTCCA	NM_053095.3
	Reverse	ATTTCTGCATCCAGGTCAGG	
Bmp7	Forward	TTTGATATCACAGCCACCAGCAAC	NM_007557.3
	Reverse	ATGGACTTCCGTGGCCTTGAAGAA	
Cxcl3	Forward	CAGCCACACTCCAGCCTA	NM_203320.3
	Reverse	CACAACAGCCCCTGTAGC	
Cxcl12	Forward	TGCACGGCTGAAGAACAACAACAG	NM_013655.4
	Reverse	TCACACCTCTCACATCTTGAGCCT	
Ccl11	Forward	CAGATGCACCCTGAAAGCCATA	NM_011330.3
	Reverse	TGCTTTGTGGCATCCTGGAC	
Cxcl5	Forward	GCCGCTGGCATTTCTGTTG	NM_009141.3
	Reverse	GCAAACACAACGCAGCTCC	
Gapdh	Forward	TGCACCACCAACTGCTTAG	NM_001289726.2
	Reverse	GGATGCAGGGATGATGTTC	

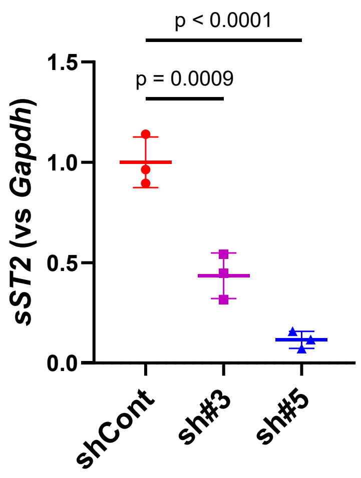

Fig. S1. RT-qPCR analysis of sST2 expression in Panc02-shCont (shCont) and Panc02-shsST2 (sh#3 ad sh#5) cells. Bars represent mean ± SD. Statistical significance was evaluated using one-way ANOVA.

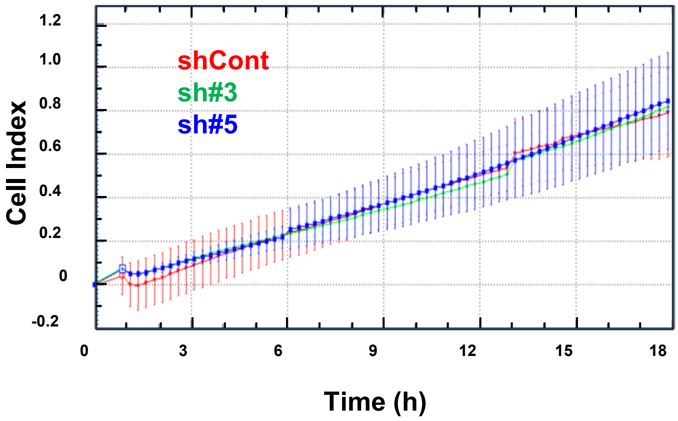

Fig. S2. In vitro Matrigel invasion assay using the xCELLigence instrument.

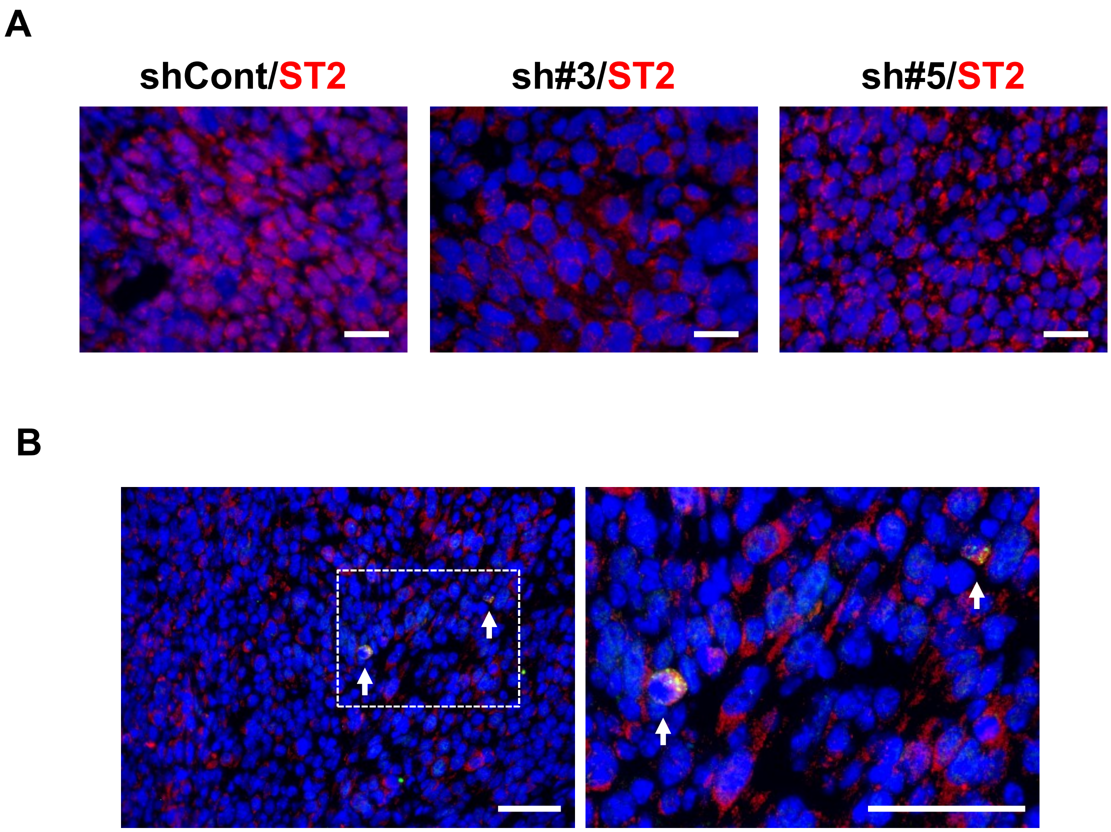

Fig. S3 Immunofluorescent analysis of the ST2 expression in Panc02 tumors. (A) ST2 expression in Panc02-shCont, -sh#3 and -sh#5 tumors. Cryosections were stained with anti-ST2 antibody which reacts with both sST2 and ST2L. Bars: 50 μm. (B) Double immunostaining with anti-ST2 antibody (red) and anti-CD90 antibody (green). The arrows indicate the $ST2^{+}CD90^{+}$ cells. Cell nuclei were stained with DAPI. Bars: 50 μm.

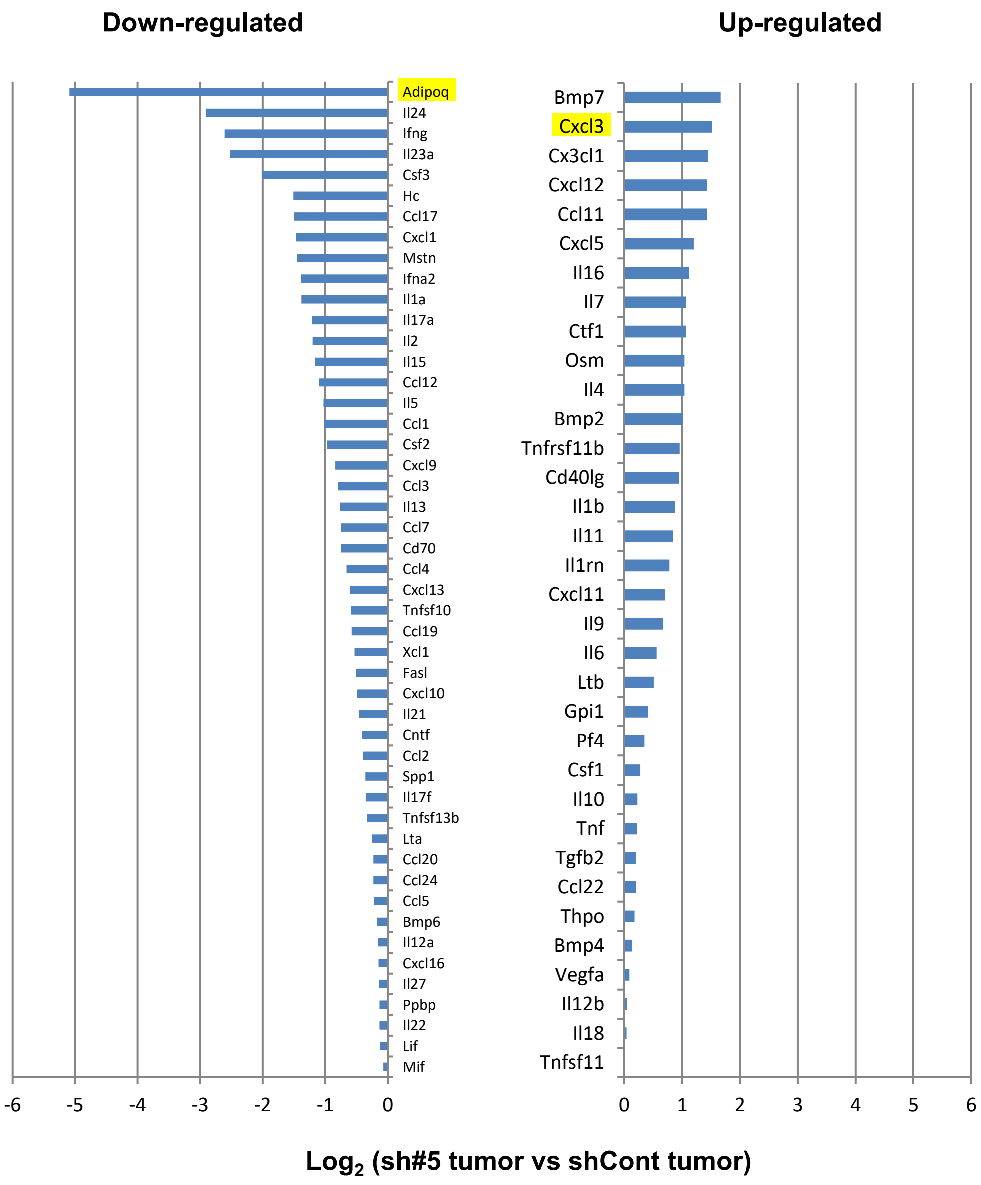

Fig. S4 PCR array analysis of cytokine and chemokine gene expression profiles in shCont and sh#5 Panc02 subcutaneous tumors.

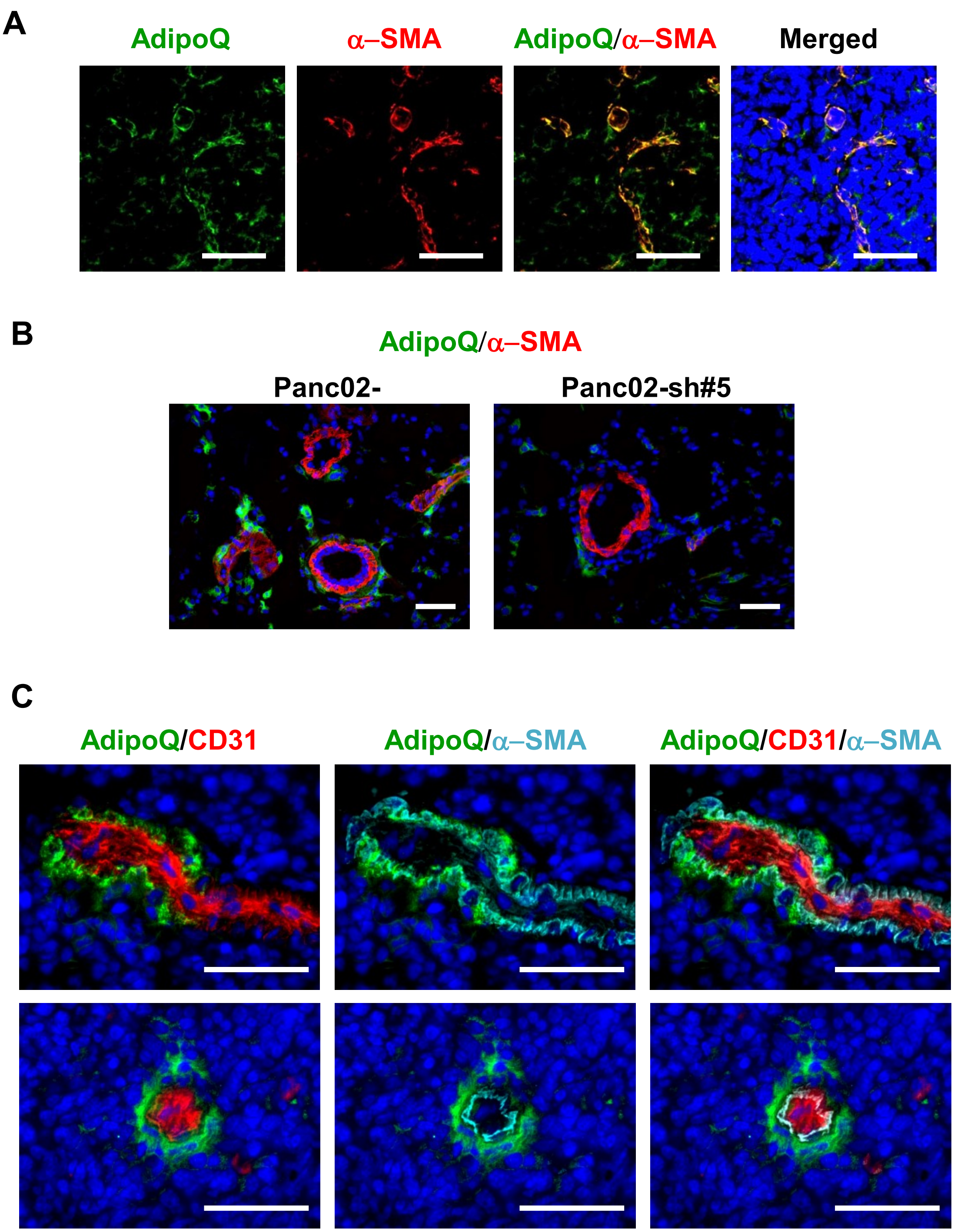

Fig. S5 Localization of AdipoQ near microvessels. (A) Tumor sections of Panc02–shCont tumors were double immunostained for AdipoQ and α-SMA. Bars: 100 μm. (B) Tumor sections of Panc02–shCont and Panco2–sh#5 tumors were double immunostained for AdipoQ and α-SMA. Bars: 50 μm. (C) Tumor sections of Panc02–shCont tumors were triple immunostained for AdipoQ, CD31 and α-SMA. Cell nuclei were stained with DAPI. Bars: 50 μm.

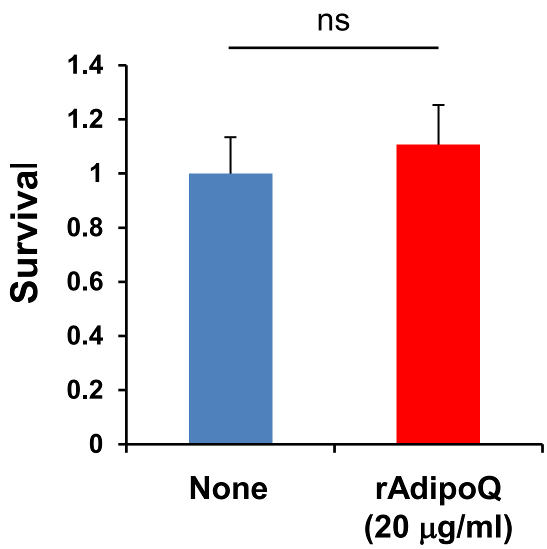

Fig. S6 Effect of rAdipoQ on the survival of Panc02-shCont cells. The cells were treated with rAdipoQ (20 μg/mL) for 2 days. An MTT assay was used to evaluate cell viability. (n=3). Bars represent SDs. Statistical significance was evaluated using Student's t-test. ns: not statistically significant.

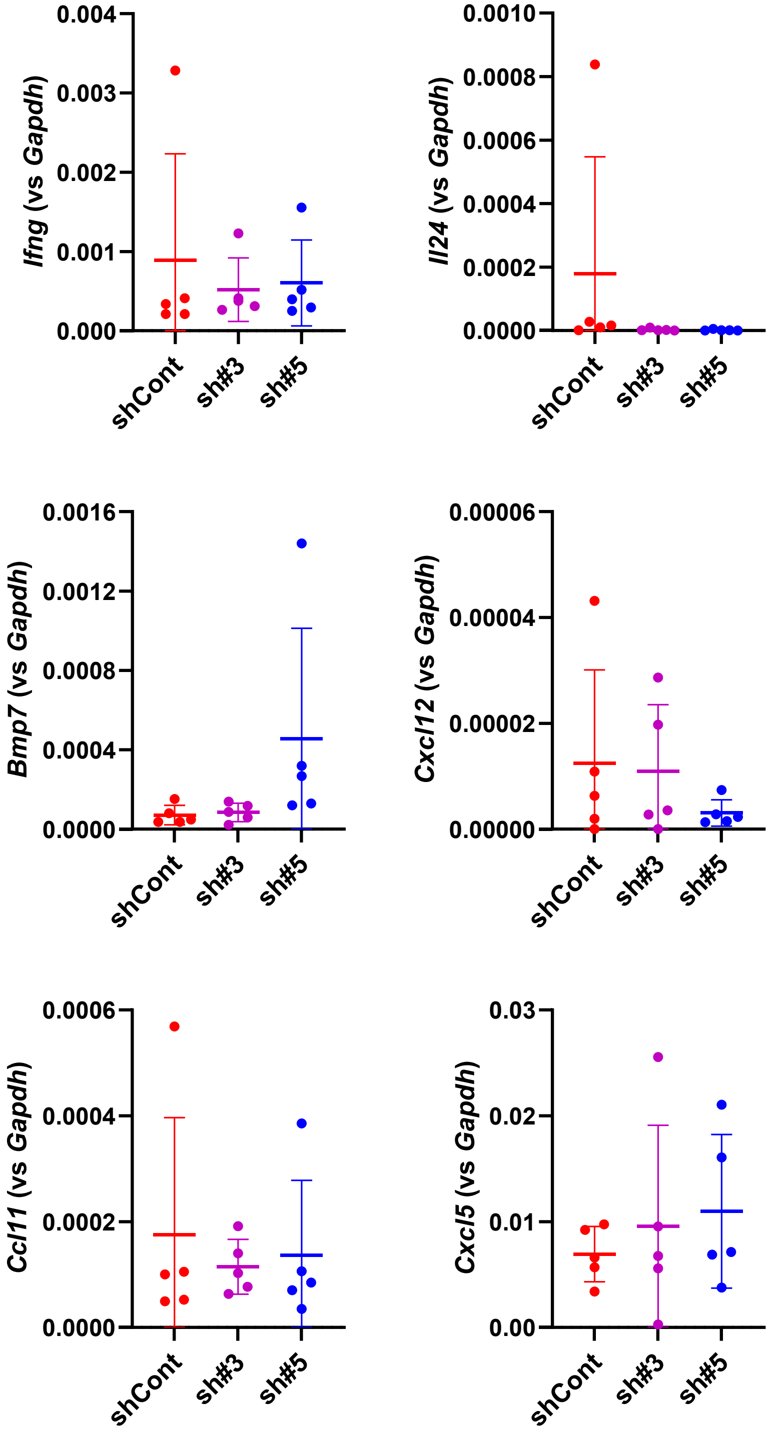

Fig. S7 RT-qPCR analysis of the expression of the indicated genes in the indicated subcutaneous tumors (n = 5). Bars represent mean ± SD.

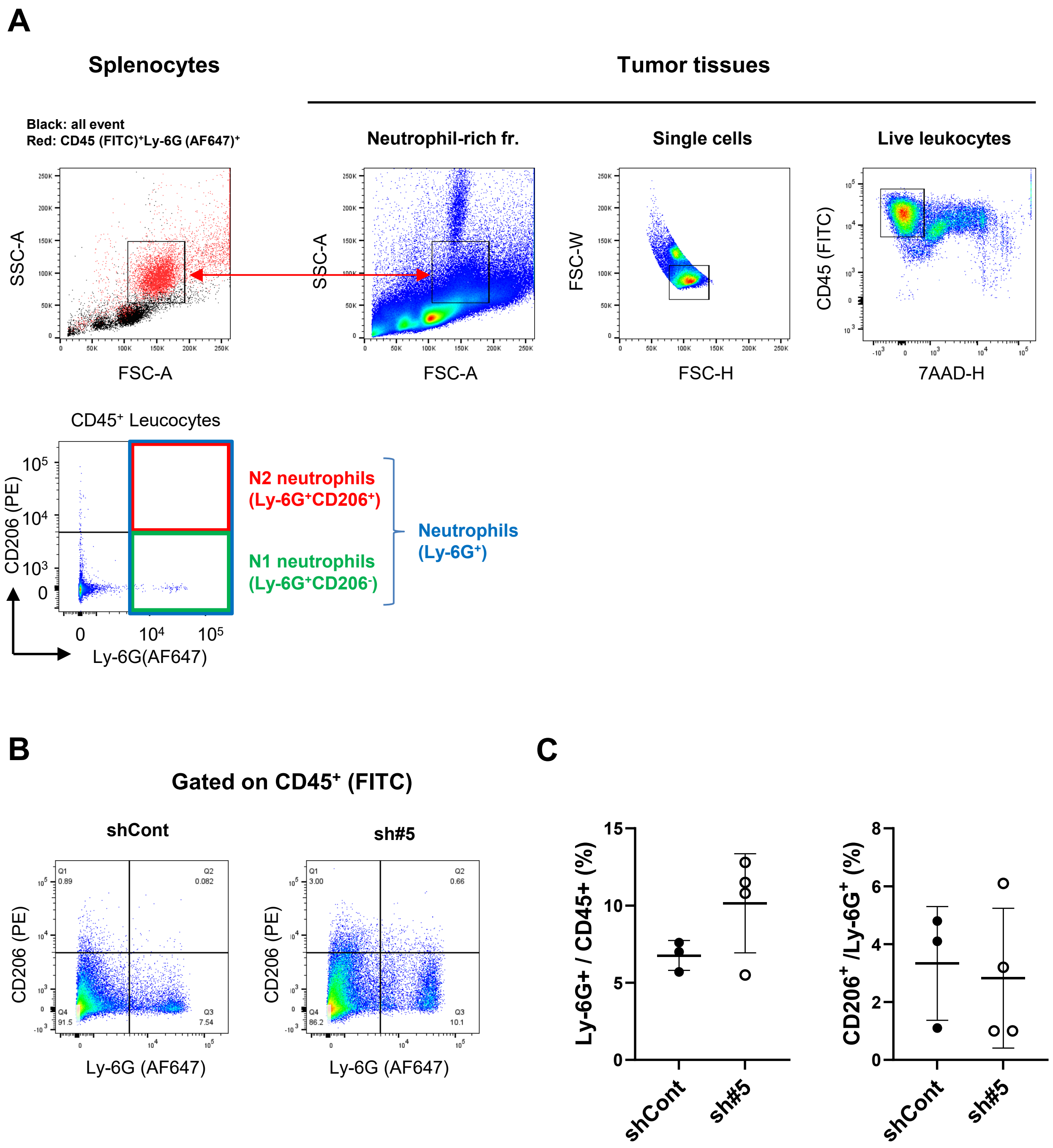

Fig. S8 Flow cytometry analysis of neutrophils in Panc02–shCont and Panc02–sh#5 tumors. (A) Flow cytometry gate setting. CD45⁺Ly-6G⁺ cell population area in splenocytes was applied to single cell population from tumor tissues. (B) Flow cytometry of CD45⁺ leucocytes in Panc02–shCont and Panc02–sh#5 tumor tissues. (C) The percentage of Ly-6G⁺ neutrophils in CD45⁺ leukocytes and CD206⁺ neutrophils in Ly-6G⁺ neutrophils in Panc02–shCont (n=3) and Panc02–sh#5 tumor tissues (n=4). Bars represent mean ± SD.

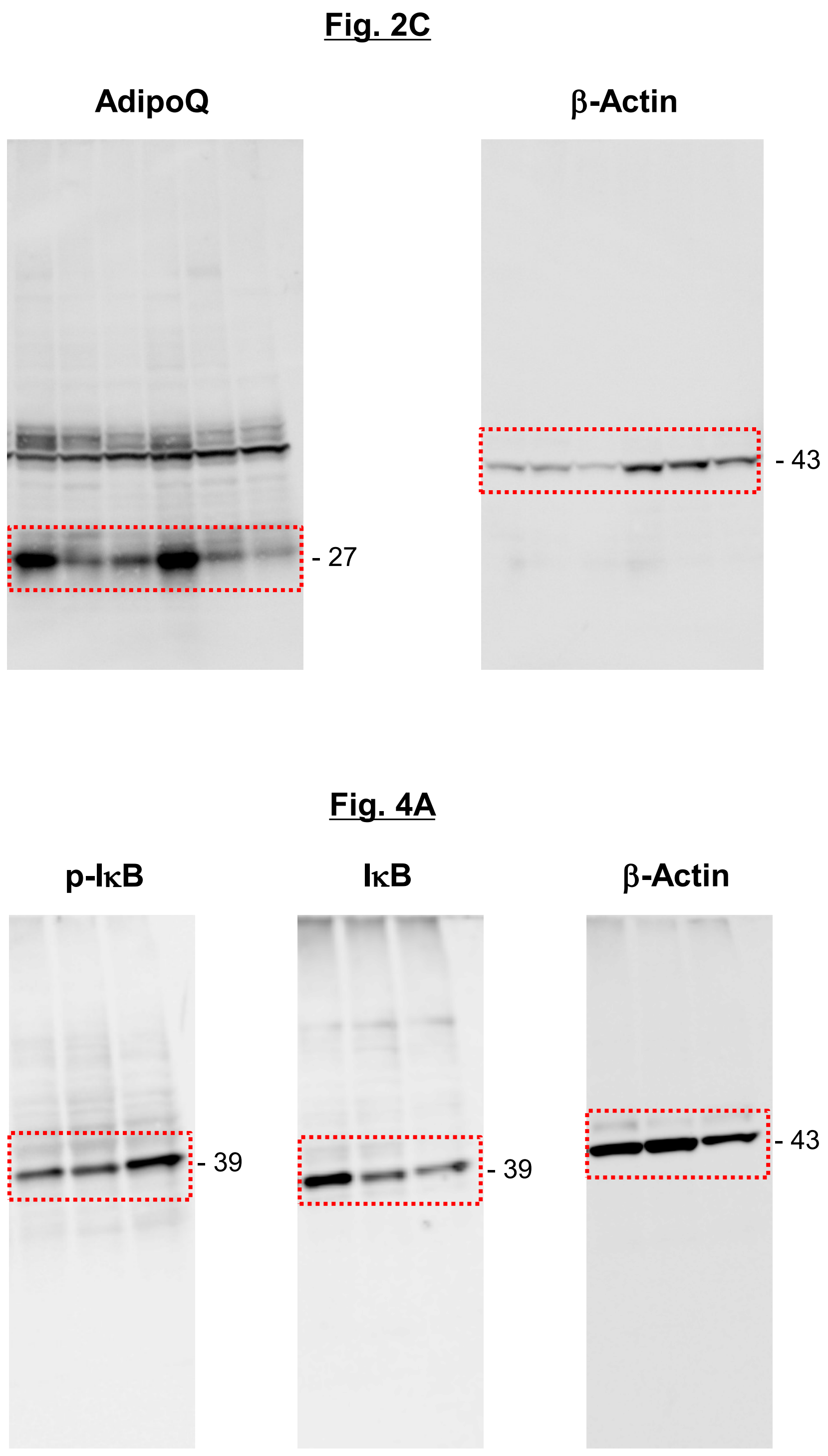

Fig. S9. Uncropped western blot images for Fig. 2C and Fig. 4A.
